# Manipulation Technique for Precise Transfer of Single Perovskite Nanoparticles

**DOI:** 10.3390/nano10071306

**Published:** 2020-07-03

**Authors:** Filipp Komissarenko, George Zograf, Sergey Makarov, Mikhail Petrov, Ivan Mukhin

**Affiliations:** 1 Department of Physics and Engineering, ITMO University, Kronverksky pr. 49, St. Petersburg 197101, Russia; g.zograf@metalab.ifmo.ru (G.Z.); s.makarov@metalab.ifmo.ru (S.M.); m.petrov@metalab.ifmo.ru (M.P.); imukhin@yandex.ru (I.M.); 2 Laboratory of renewable energy sources, St. Petersburg Academic University, Khlopina st. 8/3, St. Petersburg 194021, Russia

**Keywords:** nanofabrication, nanomanipulation, perovskites, barium titanate

## Abstract

In this article, we present the pick-and-place technique for the manipulation of single nanoparticles on non-conductive substrates using a tungsten tip irradiated by a focused electron beam from a scanning electron microscope. The developed technique allowed us to perform the precise transfer of single BaTiO_3_ nanoparticles from one substrate to another in order to carry out measurements of elastic light scattering as well as second harmonic generation. Also, we demonstrate a fabricated structure made by finely tuning the position of a BaTiO_3_ nanoparticle on top of a dielectric nanowaveguide deposited on a glass substrate. The presented technique is based on the electrostatic interaction between the sharp tungsten tip charged by the electron beam and the nanoscale object. A mechanism for nanoparticle transfer to a non-conductive substrate is proposed and the forces involved in the manipulation process are evaluated. The presented technique can be widely utilized for the fabrication of nanoscale structures on optically transparent non-conductive substrates, which presents a wide range of applications for nanophotonics.

## 1. Introduction

For the last decade, studies concerning nonlinear optical properties of non-plasmonic structures are of great interest for researchers due to the active development of nanophotonics associated with the use of dielectric materials (all-dielectric photonics). Bulk nonlinearity of dielectric and semiconductor materials offers low losses and an enhanced nonlinear response in comparison with metals [1,2,3,4,5]. Barium titanate BaTiO_3_ (BTO), a ferroelectric perovskite, is among materials which support high second-order nonlinear generation [6,7]. BTO nanostructures and especially single BTO nanoparticles, due to supporting Mie resonances in the visible range, are of great interest to second harmonic generation (SHG) studies [8,9,10,11,12].

For the study of single BTO nanoparticles, as well as for the fabrication of complex nanostructures based on them, the use of nanomanipulation methods can be in high demand as such methods can be used to successfully tackle the problems of studying a specific pre-selected object, distributing objects on a pre-patterned substrate in a certain way, transferring objects from the initial substrate to the auxiliary substrate at a predefined position, and forming assemblies from various objects. The fact that the dielectric constant of the substrate on which the dielectric nanoparticle is located can affect its optical properties [13,14] makes methods that allow manipulating nanoparticles on non-conductive substrates especially in demand.

The manipulation of a single nanoobject can be implemented using various microscopes and can be based on various mechanisms of interaction between a manipulated object and a manipulating tool. It should be noted that methods which allow picking up, transferring, and dropping a single particle from one substrate to another maintain significant research interest. All methods that allow manipulating individual micro- and nano-scale objects fall into the following groups: optical tweezers [15], manipulation via scanning probe microscopes (SPM) [16,17,18,19,20,21], and manipulation via scanning electron microscopes (SEM) [22,23,24,25,26,27] or transmission electron microscopes (TEM) electron tweezers [28,29].

However, these methods have certain limitations. The use of optical and electronic tweezers can be implemented only in a specific environment. Meanwhile, SPM-based manipulation has a lot of mechanisms of interaction between the probe and the manipulation object, including mechanical [16,17,18], electrostatic [18,19], and chemical [20,21]. 

Despite the wide range of possibilities, manipulation in SPM has a significant limitation in that the same probe is used both for particle manipulation and for visualization of the result. Thus, this strategy is based on the sequential movement of the particle and the subsequent visualization of the result of the movement instead of in-situ visualization. Another drawback of such methods is that SPM does not allow for the visualization of complex structures or three-dimensional objects of complex geometry.

SEM overcomes these limitations and makes it possible to visualize, and therefore control the process of manipulation in real-time. Built-in SEM chamber manipulators with grips [22,23] and sharpened tips [24,25,26] allow the implementation of mechanical [22,23,24,25] or electrostatic [26,27] mechanisms of interaction between the manipulating object and the manipulating tool. However, despite the advantage over SPM, the use of electron beam based techniques is limited for non-conductive substrates.

This article presents a technique that allows single nanoparticles to be transferred with high accuracy to a given area on non-conductive, optically transparent substrates while controlling the transfer process in real-time. We demonstrate experimental results of particle transfer from both conductive and non-conductive initial substrates to an auxiliary non-conductive substrate in order to study the optical properties of single BTO nanoparticles and fabricate complex all-dielectric nanophotonic structures. This technique is a further development of the approach demonstrated in [27] and successfully implemented in [30,31,32,33], and is based on the combination of a mechanical manipulator and electrostatic impact on the object of manipulation.

## 2. Materials and Methods

The manipulation experiments were carried out with a Zeiss Neon 40 EsB Scanning Electron Microscope with a Kleindiek Nanotechnik MM3A-EM piezo-driven micromanipulator installed in the chamber. The micromanipulator allows mechanical motion of the manipulator’s arm in the area under the microscope column in three axes with positional accuracy of about 10 nm. A sharp tungsten tip was attached to the manipulator’s arm using epoxy resin, which provides electrical insulation. The sharpening of the tips was carried out by electrochemical etching of 150-µm diameter tungsten wire. The etching process was carried out with an etching machine also used for scanning tunnel microscopy tip preparation [34]. Moreover, the use of experimentally verified optimal etching parameters ensured the reproducibility of the obtained tips with a radius of curvature of about 100 nm. The low cost and simplicity of creating sharped tips is a great advantage of the method since it allows quick replacement of a damaged tip with a new one.

**Figure 1:**
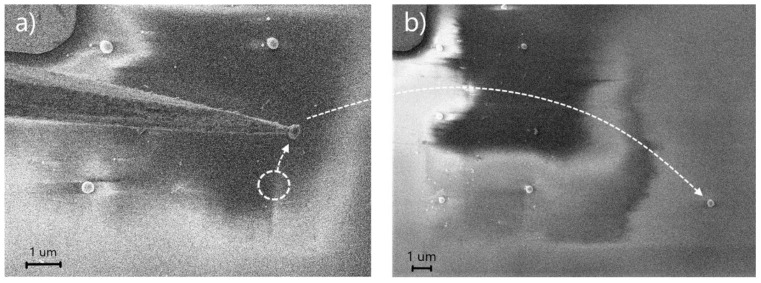
The process of manipulation of a single silicon nanoparticle on a glass substrate. (**a**) a tungsten tip with an attached silicon nanoparticle 250 nm in diameter from the glass; (**b**) the result of the transfer of the picked-up particle from the tungsten tip back to the surface of the substrate.

Silicon nanoparticles with a diameter of 250 nm were used as test objects in order to demonstrate the possibility and accuracy of the technique. Particles were produced on a glass substrate by means of laser ablation [35]. The following electron microscope operating parameters were used during manipulation: accelerating voltage of 2 kV and beam current of 10 pA. These parameters also enabled minimization of the negative effects of excess charge on a non-conductive substrate with an electron beam and allow for the observation of the position of the tip and particle with a resolution sufficient to control the process. After positioning a selected nanoparticle in the SEM, a tungsten tip was introduced into the SEM scanning area. After the tip touched the particle, the manipulator was moved upward from the substrate surface and the particle stayed on the tip. The attached nanoparticle could then be transferred to a certain area and dropped (see Figure 1). Particle dropping from the tip was performed by reintroducing the nanoparticle with the substrate in the predefined area and moving the manipulator tip to the side. 

The use of the microscope operating mode with reduced accelerating voltage and current, as well as the use of a non-conductive substrate significantly affect the composition and magnitude of forces in the tip-particle and tip-particle-substrate systems. We will consider the mechanism of picking up a dielectric particle from a non-conductive substrate and its subsequent position onto a non-conductive substrate. The manipulating mechanism presented in this article is based on the electrostatic manipulation approach presented in [27].

**Figure 2:**
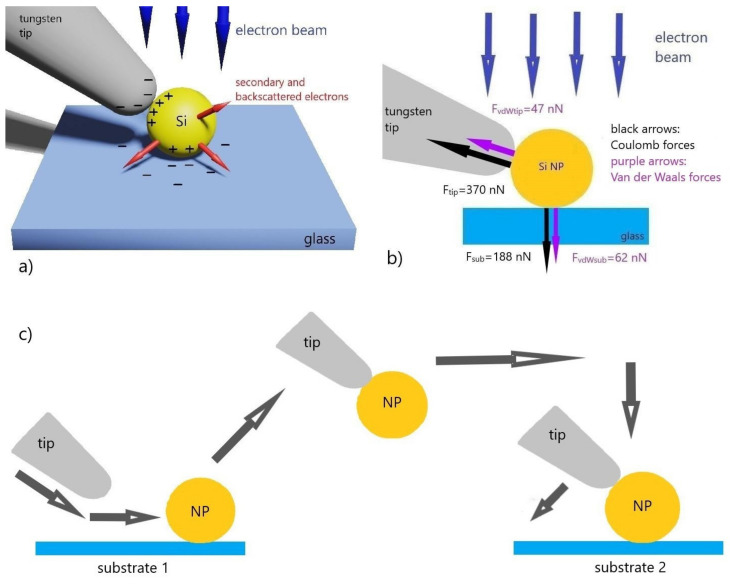
(**a**) The mechanism of charging by a scanning electron beam. The substrate is negatively charged by the primary electron beam while, due to the spherical shape, the nanoparticle is positively charged. At the particle-substrate interface a double electric layer is formed, meanwhile an ungrounded metal tip is negatively charged by the primary electron beam. When the tip touches the positively charged particle, a double electric layer with a higher capacitance, compared to the particle-substrate interface, is formed; (**b**) Diagram of forces acting on a nanoparticle at particle-substrate and tip-particle interfaces; (**c**) The scheme of the particle retrieval at the manipulator tip, moving to the other substrate, and subsequent release. The grey arrows depict the direction of tip movement.

The electron beam with an acceleration voltage of a 2 kV negatively charges the dielectric substrate. Meanwhile, due to the spherical shape of the particle, emission of secondary electrons is increased and the electron beam, under certain conditions, can charge the particle positively [36]. The positive charge accumulated within the particle is localized at the point of contact with the substrate. At the same time, a double electric layer is formed at the particle-substrate interface such that the particle is held on the substrate not only by van der Waals forces but also by an additional Coulomb electrostatic force (Figure 2a). An electrically isolated tungsten tip is charged negatively by an electron beam to a threshold at which field emission begins from the tip and the current of that field emission does not exceed the microscope beam current.

When a charged tip is put into contact with a particle another double electric layer with a large number of charges is formed since the tip-particle interface has a larger capacitance compared to the particle-substrate interface [37] (Figure 2b).

As a result, when the tip and the particle are in contact, electrostatic forces at the particle-tip interface exceed the electrostatic forces at the particle-substrate interface. Hence, when the tip is touching the nanoparticle, the sum of the forces acting on the particle from the tip can be greater than the sum of the forces acting from the substrate. Therefore, the particle stays on the tip when the manipulator is raised upward from the substrate. After picking up the particle, a double electric layer holding it to the tip in addition to the van der Waals force remains at the tip-particle interface. To drop a particle in a certain area it is necessary to create a particle-substrate interface by placing it in a selected area on the substrate and then to destroy the tip-particle interface by moving the manipulator tangentially towards the substrate. This leads to a substantial decrease in the electromagnetic forces between the tip and the particle while forces acting from the substrate remain constant, resulting in the particle effectively repositioned on the substrate. Figure 2c shows the scheme and tip movement trajectory of single particle pick-and-place.

## 3. Results and Discussion

Based on the mechanism proposed above, a numerical simulation of the pick-up of a spherical silicon particle with a diameter of 250 nm by a tungsten tip with a radius of curvature of 100 nm from the surface of the glass substrate was carried out and the emerging forces were estimated. The electrostatic forces acting on the particle from the tip and substrate were calculated using the virtual force method [38] implemented in the Ansoft Maxwell software. To determine these forces, it was necessary to estimate the maximum charges formed on the metal tip, the particle, and the substrate on irradiation by an electron beam. 

According to our assumption, the tip will be charged by the electron beam until the field emission current is equal to the microscope’s beam current. In this case, the charge will form an inhomogeneous electrostatic field with a maximum intensity of *Emax*. Integrating of the Fowler–Northheim equation [39], which relates the value of electrostatic field intensity to the field emission current density through the material’s work function, one can find the value of the net charge, which creates a field with the maximum intensity *Emax* near the tip within certain geometrical parameters. For a tip with a radius of 100 nm, the charge was 0.1 pC, while the maximum value of the electrostatic field strength *Emax* was equal to 1.8 × 10^9^ V/m, which is in good agreement with typical field values during field emission [40]. This approach was also used to determine the net charge of the particle, which amounted to 5 × 10^−17^ C. 

The charge accumulated in the substrate during irradiation by the beam was estimated using the expressions presented in [41] and amounted to 0.7 pC for the scanning area of the beam with an area of 100 μm^2^. The van der Waals forces between the particle and the substrate and between the tip and the particle were calculated using the expressions from [42,43]. In this case, a Hamaker constant of 26.8 × 10^−20^ J for the particle–substrate interface and 45.2 × 10^−20^ J for the tip–particle interface were used. The distance between the contacting surfaces was taken as 0.3 nm taking into account a monolayer of water separating the particle from the substrate and which is still preserved under vacuum [19]. The van der Waals forces were 62 nN for the substrate-particle interface and 47 nN for the tip-particle interface. 

Using the obtained values of the charges, we determined the electrostatic forces acting on the particle-substrate interface as *Fsub* = 188 nN and on the particle-tip interface as *Ftip* = 370 nN by the finite element method. Based on the obtained values, it can be concluded that when the tip touches the particle, the sum of the forces acting from the side of the tip will exceed the sum of the forces holding the particle on the substrate and, therefore, the particle can be picked up. After attaching to the tip, the particle continues to be held on the tip by both Coulomb and van der Waals forces. To remove the particle from the tip, the particle should be put into contact with the substrate and the tip-particle interface should be destroyed by mechanical moving the manipulator tangentially across the surface of the particle, towards the substrate. After that, the particle will remain on the contact point because only forces acting between particle and substrate remain: Coulomb and van der Waals for non-conductive substrates and only van der Waals for conductive substrates. 

**Figure 3:**
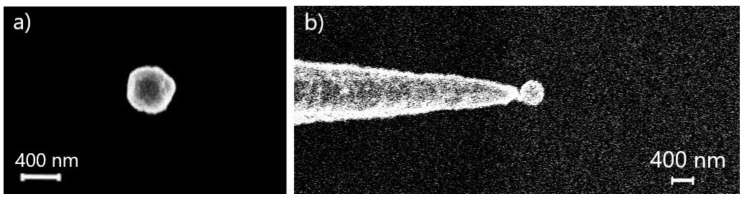
(**a**) SEM image of a single BTO particle; (**b**) a tungsten tip with a picked-up BTO particle.

The developed pick-and-place technique was used in the study of optical properties of single BTO nanoparticles. BTO particles with diameter of 420 nm were deposited from an alcohol solution onto a silicon substrate in order to select a particle with suitable size and shape in SEM (Figure 3a). After the tip was introduced into the electron beam scanning area, the selected BTO particle was first mechanically shifted from its place by the tip, picked up, and then lifted from the substrate (Figure 3b). Then, the nanoparticle was transferred to an optically transparent glass substrate covered with an indium tin oxide thin layer and dropped off to a clean area without other particles, contamination, or defects within a radius of 30 microns.

The difficulty in the manipulation process was associated with the necessity to discharge the particles without any effect on the substrate since introduced defects (such as scratches) could lead to distortion of the particle optical scattering pattern. In addition, it was necessary to prevent mechanical damage of particles during manipulation. The use of the presented manipulation method avoided both substrate and particle damage.

It should be noted that, according to the mechanism presented above, in the case of a conductive substrate, there is no electrostatic force that additionally holds the particle on the substrate and, therefore, less impact from the tip is required to pick up the particle. However, the opposite situation was observed in the experiments with BTO nanoparticles compared to Si nanoparticles. To capture a BTO particle, it was first necessary to mechanically shift the particle from its original position. This discrepancy can be explained by the two following factors. First, the SEM study showed that BTO particles are not perfectly spherical and have a more complex geometry (Figure 3a). Respectively, the BTO particle’s contact area with the substrate can be significantly larger than the contact area of silicon particles which have a more spherical shape. Thus, van der Waals forces holding the particle on the substrate will also be significantly higher for BTO particles. Secondly, the method of the particle deposition on a substrate can be crucial for the magnitude of the adhesion force [43]. For example, the precipitation of particles from a liquid suspension by droplet formation and subsequent drying can lead to condensation of impurities in the contact area [44], which also leads to an increase in adhesion forces to the substrate.

**Figure 4:**
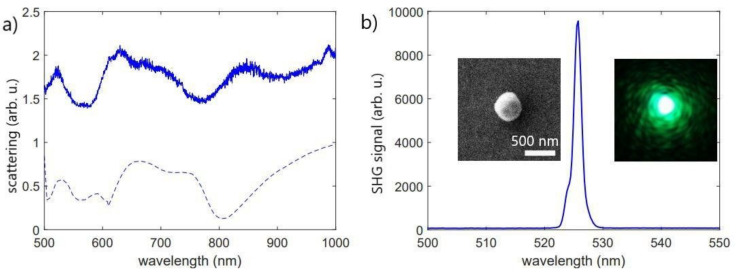
(**a**) Experimental (solid line) and simulated (dashed line) spectra of elastic scattering of light from single BTO nanoparticle; (**b**) experimental SHG spectrum for the same single BTO nanoparticle, left inset: SEM image of BTO nanoparticle; right inset: image from CCD camera.

Figure 4a depicts experimental elastic scattering of light from a single BTO nanoparticle placed on ITO substrate (SEM images are shown in the inset of Figure 4b) by the tip manipulation technique. In our experiment the halogen lamp was taken as a white light source for scattering excitation and the angle of incidence was selected as 67 degrees with respect to the normal of the substrate surface. Dark-field scattering spectra were measured in s-polarization. 

Figure 4a depicts both spectra of experimental elastic scattering of light from a single BTO nanoparticle and the corresponding simulation for the particle with the same size. Numerical calculations were carried out using the COMSOL Multiphysics 5.5 software package. One can see that the nanoparticle supports pronounced a resonant response in the scattering spectrum at 525 nm which corresponds to SHG wavelength (see Figure 4b). Moreover, one can see the resonant excitation at the fundamental 1050 nm wavelength due to the presence of a resonant mode around 1000 nm (the longer wavelengths are out of detection range of the used spectrometer). It is important to note that the simulation and experimental data are in good agreement while the slight difference between them is caused by the BTO nanoparticle used in experiments having a non-spherical shape compared to the simulated one. 

Figure 4b shows the experimental SHG spectrum for the very same nanoparticle with the excitation source of a femtosecond laser system with a central wavelength at 1050 nm and pulse duration of 150 fs (TeMa, Avesta Project). The left inset shows a SEM image of the BTO nanoparticle where the scale bar is 500 nm. The right inset corresponds to the CCD camera image obtained with sub-1 second exposure time. Presented results of optical measurements demonstrate the high efficiency of the SHG in BTO resonant nanoparticles.

**Figure 5:**
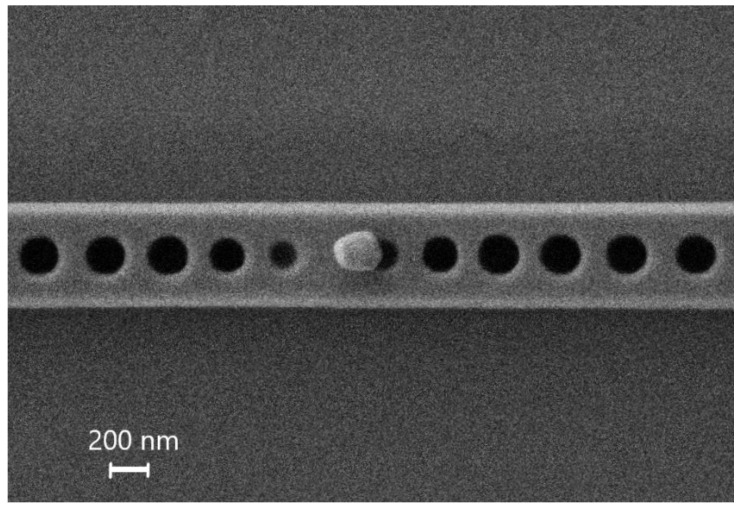
The result of the transfer of a BTO particle to a Si waveguide on a glass substrate.

The presented manipulation technique is applicable for nanofabrication of complex dielectric nanostructures. By means of the developed method, 250-nm diameter BTO nanoparticles were precisely positioned on a waveguide made of a Si strip with two groups of holes on a glass substrate (Figure 5). The single BTO particle located on the top of waveguide is considered to be a nanoantenna with a specific scattering radiation profile. Particle pick up was performed as mentioned above. Then a glass substrate, with the waveguide on it, was introduced under the electron beam. After that the tip with the particle was brought to the waveguide. When the tip with the particle touched the waveguide at a determined location, the tip was moved tangentially to the side and the particle stayed on the waveguide. The developed technique allows single nanoparticle positioning in the one-dimensional nanobeam cavity (Figure 5). The necessity for an asymmetric placement of the particle relative to the holes is dictated by the results of the numerical simulation of the nanostructure [45]. In most cases, it was possible to transfer the particle to a given region with an accuracy of approximately 50 nm. Optical measurements of the fabricated complex structures are out of the scope of current work.

## 4. Conclusions

We demonstrated the technique of electrostatic manipulation of nano-objects on non-conductive optically transparent substrates and showed the experimental results of the developed method in studies of single BTO nanoparticles as well as in the fabrication of a complex dielectric structure. This technique is implemented in SEM and is based on the electrostatic interactions of the manipulated object with an ungrounded metallic tip. A mechanism that describes the manipulation process is proposed and the forces arising upon the charging of an isolated metal tip, a dielectric nanoparticle, and a substrate by an electron beam are estimated. The presented method can be used both in the study of the essential properties of nano-objects and in the fabrication and study of new functional nanostructures for nanophotonics, nano-optomechanics, photovoltaics, and material studies.

## References

[1] Jahani S., Jacob Z. (2016). All-dielectric metamaterials. Nat. Nanotechnol..

[2] Krasnok A.E., Miroshnichenko A.E., Belov P.A., Kivshar Y.S. (2012). All-dielectric optical nanoantennas. Opt. Express.

[3] Savelev R.S., Makarov S.V., Krasnok A.E., Belov P.A. (2015). From optical magnetic resonance to dielectric nanophotonics (A review). Opt. Spectrosc..

[4] Kuznetsov A.I., Miroshnichenko A.E., Brongersma M.L., Kivshar Y.S., Luk’yanchuk B. (2016). Optically resonant dielectric nanostructures. Science.

[5] Kruk S., Kivshar Y. (2017). Functional Meta-Optics and Nanophotonics Governed by Mie Resonances. ACS Photonics.

[6] Lu H.A., Wills L.A., Wessels B.W., Lin W.P., Zhang T.G., Wong G.K., Neumayer D.A., Marks T.J. (1993). Secondharmonic generation of poled BaTiO_3_ thin films. Appl. Phys. Lett..

[7] Lin P.T., Wessels B.W., Jang J.I., Ketterson J.B. (2008). Highly efficient broadband second harmonic generation using polydomain epitaxial barium titanate thin film waveguides. Appl. Phys. Lett..

[8] Kim E., Steinbruck A., Buscaglia M.T., Buscaglia V., Pertsch T., Grange R. (2013). Second-harmonic generation of single BaTiO_3_ nanoparticles down to 22 nm diameter. ACS Nano.

[9] Ma C., Yan J., Wei Y., Liu P., Yang G. (2017). Enhanced second harmonic generation in individual barium titanate nanoparticles driven by Mie resonances. J. Mater. Chem. C.

[10] Timpu F., Hendricks N.R., Petrov M., Ni S., Renaut C., Wolf H., Isa L., Kivshar Y., Grange R. (2017). Enhanced second-harmonic generation from sequential capillarity-assisted particle assembly of hybrid nanodimers. Nano Lett..

[11] Pu Y., Grange R., Hsieh C.L., Psaltis D. (2010). Nonlinear optical properties of core-shell nanocavities for enhanced second-harmonic generation. Phys. Rev. Lett..

[12] Timpu F., Sergeyev A., Hendricks N.R., Grange R. (2017). Second-harmonic enhancement with Mie resonances in perovskite nanoparticles. ACS Photonics.

[13] Markovich D.L., Ginzburg P., Samusev A.K., Belov P.A., Zayats A.V. (2014). Magnetic dipole radiation tailored by substrates: Numerical investigation. Opt. Express.

[14] Xifre-Perez E., Shi L., Tuzer U., Fenollosa R., Ramiro-Manzano F., Quidant R., Meseguer F. (2013). Mirror-image-induced magnetic modes. ACS Nano.

[15] Ashkin A. (1970). Acceleration and trapping of particles by radiation pressure. Phys. Rev. Lett..

[16] Sitti M., Hashimoto H. (2000). Controlled pushing of nanoparticles: Modeling and experiments. IEEE ASME Trans. Mechatron..

[17] Kim S., Shafiei F., Ratchford D., Li X. (2011). Controlled AFM manipulation of small nanoparticles and assembly of hybrid nanostructures. Nanotechnology.

[18] Decossas S., Mazen F., Baron T., Bremond G., Souifi A. (2003). Atomic force microscopy nanomanipulation of silicon nanocrystals for nanodevice fabrication. Nanotechnology.

[19] Grobelny J., Tsai D.H., Kim D.I., Pradeep N., Cook R.F., Zachariah M.R. (2006). Mechanism of nanoparticle manipulation by scanning tunnelling microscopy. Nanotechnology.

[20] Ducker W.A., Senden T.J., Pashley R.M. (1991). Direct measurement of colloidal forces using an atomic force microscope. Nature.

[21] Mukhin I.S., Fadeev I.V., Zhukov M.V., Dubrovskii V.G., Golubok A.O. (2015). Framed carbon nanostructures: Synthesis and applications in functional SPM tips. Ultramicroscopy.

[22] Mølhave K., Wich T., Kortschack A., Bøggild P. (2006). Pick-and-place nanomanipulation using microfabricated grippers. Nanotechnology.

[23] Cagliani A., Wierzbicki R., Occhipinti L., Petersen D.H., Dyvelkov K.N., Sukas Ö.S., Herstrøm B.G., Booth T., Bøggild P. (2010). Manipulation and in situ transmission electron microscope characterization of sub-100 nm nanostructures using a microfabricated nanogripper. J. Micromech. Microeng..

[24] Meyer E., Braun H.G. (2008). Micro-and nanomanipulation inside the SEM. J. Phys. Conf. Ser..

[25] Miyazaki H., Sato T. (1996). Pick and place shape forming of three-dimensional micro structures from fine particles. Proc. IEEE Int. Conf. Robot. Autom..

[26] Fukuda T., Arai F., Dong L. (2003). Assembly of nanodevices with carbon nanotubes through nanorobotic manipulations. Proc. IEEE.

[27] Denisyuk A.I., Komissarenko F.E., Mukhin I.S. (2014). Electrostatic pick-and-place micro/nanomanipulation under the electron beam. Microelectron. Eng..

[28] Zheng H., Mirsaidov U.M., Wang L.W., Matsudaira P. (2012). Electron beam manipulation of nanoparticles. Nano Lett..

[29] Oleshko V.P., Howe J.M. (2013). Electron tweezers as a tool for high-precision manipulation of nanoobjects. Adv. Imaging Electron Phys..

[30] Makarov S.V., Sinev I.S., Milichko V.A., Komissarenko F.E., Zuev D.A., Ushakova E.V., Mukhin I.S., Yu Y.F., Kuznetsov A.I., Belov P.A. (2018). Nanoscale generation of white light for ultrabroadband nanospectroscopy. Nano Lett..

[31] Renaut C., Lang L., Frizyuk K., Timofeeva M., Komissarenko F.E., Mukhin I.S., Smirnova D., Timpu F., Petrov M., Kivshar Y. (2019). Reshaping the Second-Order Polar Response of Hybrid Metal–Dielectric Nanodimers. Nano Lett..

[32] Lukashenko S.Y., Mukhin I.S., Komissarenko F.E., Gorbenko O.M., Sapozhnikov I.D., Felshtyn M.L., Uskov A.V., Golubok A.O. (2018). Resonant Mass Detector Based on Carbon Nanowhiskers with Traps for Nanoobjects Weighing. Phys. Status Solidi A.

[33] Kryzhanovskaya N., Polubavkina Y., Moiseev E., Maximov M., Zhurikhina V., Scherbak S., Lipovskii A., Kulagina M., Zadrianov Y., Mukhin I. (2018). Enhanced light outcoupling in microdisk lasers via Si spherical nanoantennas. J. Appl. Phys..

[34] Mukhin I., Zhukov M., Mozharov A., Bolshakov A., Golubok A. (2019). Influence of condensation enhancement effect on AFM image contrast inversion in hydrophilic nanocapillaries. Appl. Surf. Sci..

[35] Dmitriev P.A., Makarov S.V., Milichko V.A., Mukhin I.S., Gudovskikh A.S., Sitnikova A.A., Samusev A.K., Krasnok A.E., Belov P.A. (2016). Laser fabrication of crystalline silicon nanoresonators from an amorphous film for low-loss all-dielectric nanophotonics. Nanoscale.

[36] Reimer L. (1998). Electron scattering and diffusion. Scanning Electron Microscopy.

[37] Miyazaki H.T., Tomizawa Y., Saito S., Sato T., Shinya N. (2000). Adhesion of micrometer-sized polymer particles under a scanning electron microscope. J. Appl. Phys..

[38] McMeeking R.M., Landis C.M., Jimenez S.M. (2007). A principle of virtual work for combined electrostatic and mechanical loading of materials. Int. J. NonLin. Mech..

[39] Fowler R.H., Nordheim L. (1928). Electron emission in intense electric fields. Proc. R. Soc. Lond. A Math. Phys..

[40] Dyke W.P., Dolan W.W. (1956). Field emission. Adv. Electron. Electron. Phys..

[41] Cazaux J. (2004). Charging in scanning electron microscopy “from inside and outside”. Scanning.

[42] Hamaker H.C. (1937). The London—Van der Waals attraction between spherical particles. Physica.

[43] Hu S., Kim T.H., Park J.G., Busnaina A.A. (2010). Effect of different deposition mediums on the adhesion and removal of particles. J. Electrochem. Soc..

[44] Contreras-Naranjo J.C., Ugaz V.M. (2013). A nanometre-scale resolution interference-based probe of interfacial phenomena between microscopic objects and surfaces. Nat. Commun..

[45] Pin C., Cluzel B., Renaut C., Picard E., Peyrade D., Hadji E., de Fornel F. (2015). Optofluidic near-field optical microscopy: Near-field mapping of a silicon nanocavity using trapped microbeads. ACS Photonics.

